# Supplementary material for: Deletion of wfs1 Impairs Oligodendrocyte Precursor Cells Dorsal Distribution and Myelination Through the wfs1-hmgcs1 Axis in Zebrafish
Source: Biology (Basel). 2026 Aug 16;15(16):1402. doi: 10.3390/biology15161402 (PMC13510151; doi:10.3390/biology15161402)
Supplement: Supplementary file 1 [file biology-15-01402-s001.zip › File S1-Transcriptome sequencing data and differentially expressed genes (DEGs) in wfs1-- zebrafish at 5 dpf/1.GO/wfs1KOvsWT/GSEA/heat_map_corr_plot.html]

Heat map and correlation plot for deg  

Fig 1: heat\_map      
 Heat Map of the top 50 features for each phenotype in deg

  
  

Fig 2: Ranked Gene List Correlation Profile      
 Ranked list correlations for deg

  
  
    
